# ClipCrop: a tool for detecting structural variations with single-base resolution using soft-clipping information

**DOI:** 10.1186/1471-2105-12-S14-S7

**Published:** 2011-12-14

**Authors:** Shin Suzuki, Tomohiro Yasuda, Yuichi Shiraishi, Satoru Miyano, Masao Nagasaki

**Affiliations:** 1 Human Genome Center, Institute of Medical Science, University of Tokyo, 4-6-1 Shirokanedai, Minatoku, Tokyo, 108-8639, Japan

## Abstract

**Background:**

Structural variations (SVs) change the structure of the genome and are therefore the causes of various diseases. Next-generation sequencing allows us to obtain a multitude of sequence data, some of which can be used to infer the position of SVs.

**Methods:**

We developed a new method and implementation named ClipCrop for detecting SVs with single-base resolution using soft-clipping information. A soft-clipped sequence is an unmatched fragment in a partially mapped read. To assess the performance of ClipCrop with other SV-detecting tools, we generated various patterns of simulation data – SV lengths, read lengths, and the depth of coverage of short reads – with insertions, deletions, tandem duplications, inversions and single nucleotide alterations in a human chromosome. For comparison, we selected BreakDancer, CNVnator and Pindel, each of which adopts a different approach to detect SVs, e.g. discordant pair approach, depth of coverage approach and split read approach, respectively.

**Results:**

Our method outperformed BreakDancer and CNVnator in both discovering rate and call accuracy in any type of SV. Pindel offered a similar performance as our method, but our method crucially outperformed for detecting small duplications. From our experiments, ClipCrop infer reliable SVs for the data set with more than 50 bases read lengths and 20x depth of coverage, both of which are reasonable values in current NGS data set.

**Conclusions:**

ClipCrop can detect SVs with higher discovering rate and call accuracy than any other tool in our simulation data set.

## Background

Structural variations (SVs) are polymorphisms that change the structure of the genome, e.g. deletions, insertions, translocations, inversions and tandem duplications [1]. They induce functional change in genes and regulatory regions, which can cause various diseases [2], e.g. autism [3], Parkinson's disease [4], schizophrenia [5]. Not only inherited SVs, but also somatic SVs can be responsible for various diseases including cancer [6]. However, until a few years ago, there were no efficient methods to detect genome wide SVs in high resolution. One of the microarray analyses, array-CGH, can only detect limited SVs, since this approach can neither detect small size SVs nor clarify the single nucleotide level sequence of the target sample. Recently, next-gen sequencing (NGS) has drastically changed this situation. NGS enables us to measure large number of short digested sequence reads (short reads around 50 to 120 bases) with short time with at once [7]. Additionally, alignments of sequenced reads to the reference genome, which were impossible using the microarray approach, are now applicable. Thus, we can detect SVs with higher resolution.

Until now, three types of methods have been developed to detect SVs from NGS data: discordant pair approach, depth of coverage approach and split read approach [1].

The first approach, discordant pair, uses paired-end reads of NGS data, and calls SVs when the distance of two paired-end reads is discordant [8]. When SVs occur, paired-end reads generated from these locations cannot be mapped to the reference in concordant distance. BreakDancer [9], VariationHunter [10], MoDIL [11] and ABI Tools [12] can be categorized into this method. This idea has been developed in the early times when the depth of coverage was low and the length of the read sequences (read lengths) was short. Thus, this method is appropriate for smaller datasets of short-read. However, this method cannot detect SVs with shorter lengths, and it has difficulties to know the exact position of SV events.

The second approach, depth of coverage, is used in SegSeq [13], CNVnator [14] and ABITools [12]. It uses the frequency of mapped short reads or bases to each position on the reference genome. The main concept of this method is similar to array-CGH. When deletions occur, the number of mapped reads to regions in the reference genome will decrease. In contrast, in the case of duplications, the number of mapped reads to regions in the reference genome will increase. Different from the first approach, this does not require paired-end reads, while it requires high coverage and still has difficulties detecting shorter SV events.

The third approach, ‘split read’, is the method to detect SVs using unsuspected reads, which are not correctly mapped to the reference genome or remain unmapped. In general, split read approach is applicable only to paired-end reads. While it needs sufficient read lengths and depth of coverage, the method can detect SVs with single-base resolution. Reads on an SV event contain a ‘breakpoint’, a boundary of a region affected by SV and its flanking region which is the same as the reference genome. An SV is called when the same breakpoint is detected in unsuspected reads. The algorithm of detecting breakpoints varies with tools. Pindel [15] and SLOPE [16] use orphaned reads, unmapped reads whose mate were succeeded in mapping to the reference genome, as unsuspected reads. SLOPE attempts partial alignment between the either end of each unmapped read and the reference genome to obtain breakpoints. Pindel gets substrings from two different regions around the mapped mate read; one region is two fold of average insert size from 3’ end of mapped mate read and the other region is the sum of maximum deletion size and read lengths from the appropriate position. It then checks whether the unmapped read can be reconstructed by concatenating two substrings from each region.

Major mapping tools, such as the Burrows-Wheeler alignment tool, (e.g. BWA [17]) if they failed to map full length short read to reference genome, still try to map part of the short read. If the short read is mapped partially, then the information of the partial mapping is stored into a major mapping format SAM [18] as soft-clipping information. The number of soft-clipped reads is comparable to that of orphaned reads which is adopted in Pindel and SLOPE. Thus, our new method ClipCrop employs soft-clipping information and advances the third 'split read approach.' By using the boundary position between mapped sequence and the soft-clipped sequence in a clipped read, we can obtain putative breakpoints. Ideally, among these putative breakpoints, true breakpoint will be contained. We then remap soft-clipped sequences around the detected putative breakpoints and infer which type of event is really occurred at this region. The detailed method is described in Section 2. Section 3 demonstrates the comparison of ClipCrop, Pindel, SLOPE and BreakDander to various simulation data set. Section 4 details the result in Section 3.

## Methods

In the first process of ClipCrop, reads with soft-clipping information are chosen for the next analysis. The soft-clipping information is written as a CIGAR string in SAM format. Here is a sample data of CIGAR string: “31S69M” means 31 bases from the left end are clipped, and the rest 69 bases are matched.

The SAM file must be generated from paired-end mapping tools, and the mapping tool must generate a SAM file with soft-clipping information. In some mapping tools (e.g. BLAST [19], BLAT [20]), mapped result information file does not contain whole read sequence, but only mapped part of the sequence. In such cases, a generated SAM file from them contains hard-clipping, partially unmapped sequence that is not in the SEQ column in SAM format. We can convert hard-clipping information to soft-clipping information by using the original FASTQ file to put information about the original sequence of each read. As a result of partial alignment, there are some reads where both ends are soft-clipped (e.g. 14S54M36S). We ignored such reads because they don’t carry relevant information.

**Figure 1:**
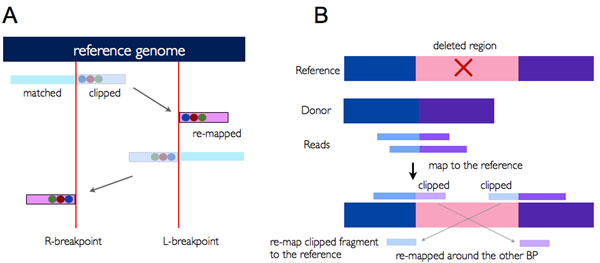
**Identification of deletion events.** (A) Relation between two breakpoints in deletion events. Clipped sequences are inside the two breakpoints, and they are remapped to outside the opposite breakpoint. (B) The way two breakpoints and clipped sequences are generated from deletion events. Reads generated from deleted region in a donor’s genome are soft-clipped, and remapped.

Second, breakpoints are obtained from the soft-clipping information. The marginal point between a clipped sequence and matched sequence is denoted as a breakpoint. When the left side of the breakpoint is clipped, it is denoted as an L-breakpoint, and R-breakpoint in the opposite case (Figure 1(A)). After identifying breakpoints, they are sorted and clustered within 5-base differences.

In the next process, soft-clipped fragments with lengths larger than 10bases are collected and remapped to the reference genome around the whole breakpoint. Before mapping, the reference genome is cut around each breakpoint with 1000-base elongation to both sides. This process can reduce the probability of clipped sequences to be mapped in the wrong position. In our current implementation, BWA is used for this remapping process. By checking the mapped pattern of clipped sequence, ClipCrop infer the SV type from deletion, inversion, tandem duplication, insertion and translocation as follows.

In deletion events, clipped sequences from an L-breakpoint are mapped to the left side of an R-breakpoint and vice versa (Figure 1). As we can see in Figure 1(B), reads generated from nearby deleted region are soft-clipped and remapped.

**Figure 2:**
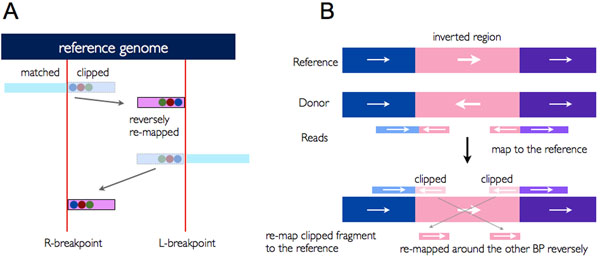
**Identification of inversion events.** (A) Relation between two breakpoints in inversion events. Clipped sequences are inside the two breakpoints, and they are remapped to outside the opposite breakpoint reversely. (B) The way two breakpoints and clipped sequences are generated from inversion events. Reads generated from inverted region in a donor’s genome are soft-clipped, and remapped reversely.

In inversion events, the same as deletion events, clipped sequence from an L-breakpoint are mapped to the left side of an R-breakpoint, but mapped reversely, and vice versa (Figure 2). Figure 2B explains the reason why the clipped sequences are mapped reversely.

**Figure 3:**
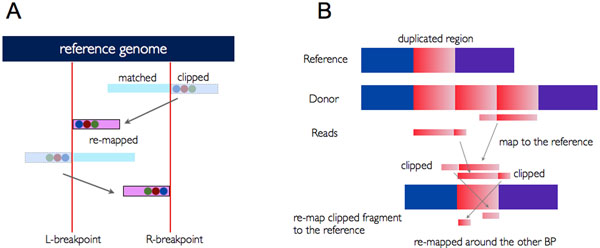
**Identification of tandem duplication events.** (A) Relation between two breakpoints in tandem duplication events. Clipped sequences are outside the two breakpoints, and they are remapped to inside the opposite breakpoint. (B) The way two breakpoints and clipped sequences are generated from tandem duplication events. Soft-clipped sequences are generated from the marginal point of two duplicated sequences.

In tandem duplication events, clipped sequences from an L-breakpoint are mapped to the right side of an R-breakpoint and vice versa (Figure 3). Unlike deletion events, clipped sequences are made outside the two breakpoints, and mapped inside the two. Soft-clipped sequences are generated from the marginal point of two duplicated sequences (Figure 3(B)).

**Figure 4:**
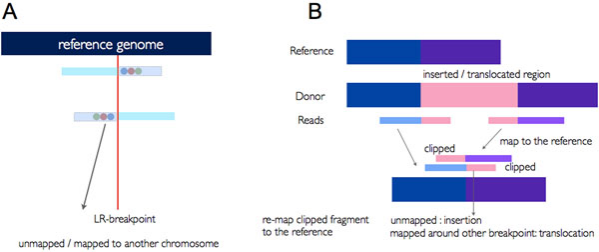
**Identification of insertion/translocation events.** (A) Relation between two breakpoints in insertion/translocation events. An L-breakpoint and an R-breakpoint are located in the same position. (B) The way two breakpoints and clipped sequences are generated from insertion/translocation events. If these clipped sequences are remapped to another region, it is a translocation event. If they remain unmapped, it is an insertion event.

In insertion and translocation, an L-breakpoint and an R-breakpoint are in the same position (Figure 4). Reads containing inserted / translocated sequence are clipped, and remapped if it is a translocation event, and unmapped if it is an insertion event.

After checking the type of SV from each soft-clipped reads, these are clustered by its type and position allowing 10 base difference at most. If two SV calls with the same type are overlapped each other, then only one SV with higher reliability score is finally selected. Reliability score is defined with the following formula:(1)

, where *B_L_* and *B_R_* are the number of clipped reads supporting the L/R-Breakpoint of the SV event, *C_L_* and *C_R_* are the number of clipped read remapped to the L-Breakpoint of the SV event. In this formula, the higher the clipped and the remapped reads, the higher the score. Also, the score tend to be high when the number of left and right reads are balanced.

## Results

We prepared various simulation data to evaluate the performance of CripCrop and other SV-detecting tools. Table 1 shows the parameter set in these simulations. In each simulation, we generated 200 SVs to the human chromosome 22 (reference build 37). The type of each SV was randomly chosen from insertion, inversion, deletion and tandem duplication (all types of SVs other than translocation). The length of each SV event followed the normal distribution determined in each data. We also set single nucleotide alteration frequency as one per 10,000 bases. After creating slightly modified chromosome 22 sequence in FASTA format, we generated paired-end short reads with FASTQ format. The depth of coverage was set to 5, 10, 15, 20 or 40 and read lengths was set to 50, 75, 100 or 108 bases. The distribution of the insert length (distance between outer positions of read pairs) was set to *N*(400, 50). In order to compare the performance of our method with other three methods - discordant-pair method, depth of coverage method and split read method -, we chose the following tools as the representative of each method; BreakDancer, CNVnator and Pindel, respectively. We removed the results of ClipCrop with its reliablity score zero.

**Table 1:** Parameters used in simulation data

Total SVs	200
Reference genome	Human chromosome 22 (Build 37 ref)
Distribution of SV length	*N*(50, 5), *N*(80, 8), *N*(100, 10), *N*(120, 12), *N*(150, 15), *N*(170, 17), *N*(200, 20), *N*(400, 40) *N*(600, 60), *N*(800, 80), ***N*****(1000, 100)** *N*(2000, 200), *N*(4000, 400)
The rate of single nucleotide alterations	1/10000
The number of tandem repeat	*N*(40, 20) (>1)
Mean depth of coverage	5, 10, 15, 20, **40**
Read lengths	50, 75, 100, **108**
Distribution of template lengths	*N*(400, 50)

We generated various data each of which has different distribution of SV length, mean depth of coverage or read lengths. Values with bold type are the default (for example, if mean depth of coverage is set to five, then the distribution of SV length and read lengths is set to *N*(1000, 100) and 108.)

**Figure 5:**
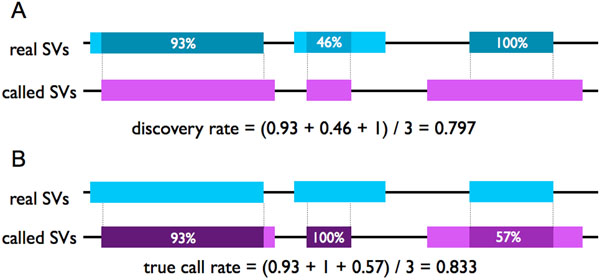
**Discovery rate and true call rate.** (A) Discovery rate is the mean of each ratio of overlapped region in the real SV between the real SV and the called SV determined by formula (2). In this case, the discovery rate is calculated as 0.797. When discovery rate is high, the number of true positive will increase. Thus, this discovery rate can be regarded as the similar concept to sensitivity. (B) True call ratio is the mean of each ratio of overlapped region in the called SV between the real SV and the called SV determined by formula (3). The true call rate is calculated as 0.833. When true call ratio is high, false positive will decrease. Thus, this true call ratio can be regarded as the similar concept to specificity.

We defined discovery rate and true call rate to compare their performance to detect true SVs (Figure 5). Let real SVs be *S_R_* = {*r*_1_ = [*x*_1_, *y*_1_], …, *r_N_*} and called SVs be *S_C_* = {*c*_1_, …, *c_M_*}, where [*x*_i_, *y*_i_] is the start and end positions of the *i*th SV. If the type of SV *r_i_* is insertion, then the inserted point *p_i_* to the reference genome is only given. In this case, we set the range of a real SV *r_i_* = [*p_i_* – 100, *p_i_* + 100]. The range of a called SV *c_i_* is defined in the same way. *Discovery rate D*(*S_R_*, *S_C_*) and *true call rate T*(*S_R_*, *S_C_*) are determined by the following formulas:(2)(3)

, where the function *F* is(4)

As tools with high sensitivity can detect with high discovery rate, it can be regarded as the similar concept to sensitivity. In the same way, true call rate can be regarded as the similar concept to specificity.

**Figure 6:**
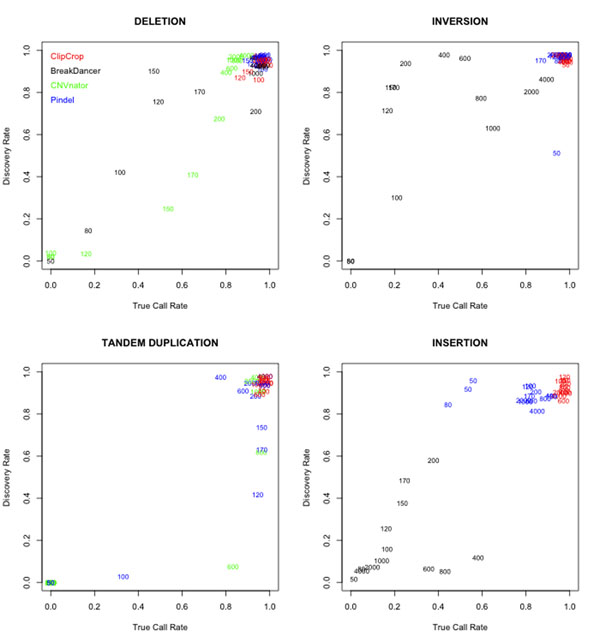
**Results 1 : discovery rate and true call rate of each method.** Discovery rates and true call rates of each data with four methods in various SVs. Numbers in graphs stand for the mean length of SVs. CNVnator only calls deletions and tandem duplications, and BreakDancer doesn’t call tandem duplication.

Figure 6 summarizes discovery rates and true call rates in each type of SVs for four tools. Integer values in the plots, e.g. 50, 80, 100, 120, 150, 170, 200, 400, 800, 1000, 2000 and 4000, represent the mean length of SVs. Because CNVnator cannot detect inversions and insertions, and BreakDancer cannot detect tandem duplication, we didn’t plot these data. In all types of SVs, ClipCrop and Pindel resulted in higher discovery rate and true call rate to the other tools. ClipCrop was better than Pindel especially to detect short tandem duplications and insertions.

**Figure 7:**
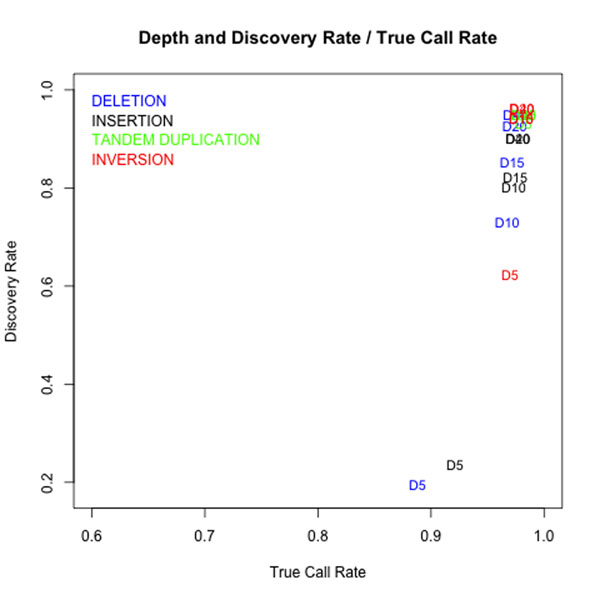
**Discovery rate / true call rate with different depth.** Discovery rates and true call rates of ClipCrop with different depth of coverages (5, 10, 15, 20, 40). Numbers in graphs stand for the mean depth of the data.

**Figure 8:**
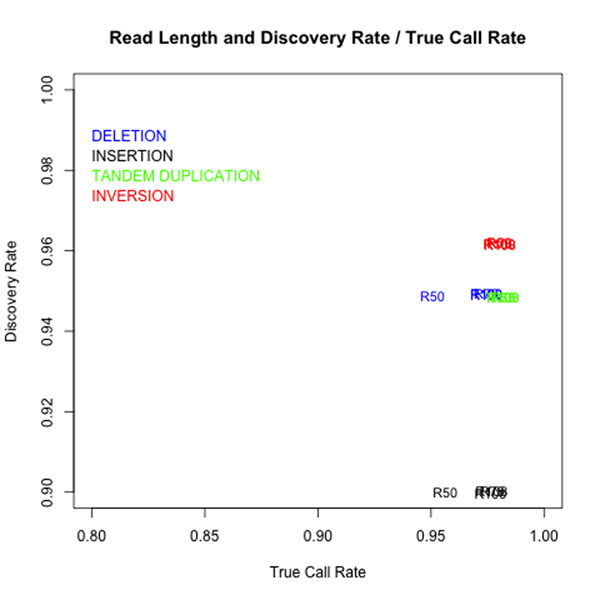
**Discovery rate / true call rate with different lengths of the read sequences.** Discovery rates and true call rates of ClipCrop with different lengths of the read sequences (50, 75, 100, 108). Numbers in graphs stand for the lengths of the data.

Figure 7 and Figure 8 respectively shows how the read lengths and the depth of coverage effects to the discovery rate and true call rate in ClipCrop. The plotted values in Figure 7, e.g. D5, D10, D15, D20 and D40, represents the depth of coverage. The plotted values in Figure 8, e.g. R50, R75, R100 and R108, denotes the read lengths. From these results, for ClipCrop the sufficient depth to detect SVs was turn out to be more than 20, and the sufficient read lengths was turn out to be more than 50.

## Discussion

In all types of SVs, ClipCrop and Pindel could detect most of SVs with high accuracy (Figure 6). It is because these two tools uses split read approach. This approach can detect SVs of any size with single-base resolution. BreakDancer, which employs discordant-pair approach, cannot detect short SVs, and its accuracy cannot be single-base resolution. CNVnator, adopting depth of coverage approach, firstly splits reference genome with a certain window size, so it cannot detect SVs with shorter length than the window size. As we set the window size to 100 bases in our analyses, CNVnator couldn’t detect SVs with length <100 bases. The resolution in CNVnator is also limited to the window size. As well as ClipCrop, Pindel also marked high discovery rate and true call rate, but it couldn’t detect short duplications ( <170 bases). This is because of the following reason. Pindel tries to reconstruct split reads by concatenating two subsequences generated from two regions near the position of mapped mate. In short duplications, reads from duplicated region would contain more than two breakpoints, which means it requires more than three subsequences to reconstruct. Thus, Pindel cannot generate these reads and fails to detect short duplications. ClipCrop, on the other hand, uses only soft-clipped sequences. Some of the short soft-clipped sequences don’t contain any breakpoints, and they can remap and support tandem duplication calls. ClipCrop also excelled over Pindel in true call rate of insertions. As formula of reliability score (1) shows, ClipCrop sets zero to SVs called from only one-side clipping and only one breakpoint, i.e. (*B_L_*, *B_R_*, *C_L_*, *C_R_*) = (*n*, 0, *m*, 0) or (*B_L_*, *B_R_*, *C_L_*, *C_R_*) = (0, *n*, 0, *m*). Thus, by removing SVs with score zero, we can obtain reliable SVs with both-side supported, which is thought to contribute its higher accuracy.

The results in Figure 7 shows that ClipCrop could detect tandem duplications with high discovery rate and true call rate even the depth of coverage is 5. This is because the depth of tandem duplicated regions is much higher than surroundings, and there are sufficient numbers of reads which support tandem duplications. Also, as inversions can be supported by twice as many reads as deletion and insertion (reads mapped to inverted region with soft-clipping also supports breakpoints), discovery rate and true call rate were higher than those of deletion and insertion. The discovery rate and true call rate were saturated at depth 20, therefore the sufficient depth for ClipCrop is turned out to be 20, which is not so high in current NGS data.

From the results in Figure 8, the sufficient read lengths for ClipCrop is more than 50 bases. Thus, ClipCrop can be applied to most of current NGS data.

There is another recently published SV-detecting tool called CREST [21], which also uses soft-clipping information. Unlike ClipCrop, CREST cannot detect tandem duplications. CREST assembles soft-clipped sequences, and remaps the assembled sequence. Thus, assembled reads from the region of tandem duplications cannot be mapped to the original reference genome.

Currently, as ClipCrop is focusing only on soft-clipping information, it doesn’t calculate the length of insertion. However, as ClipCrop calls the position of insertion with high accuracy (Figure 6), we will easily be able to obtain these information by combination of other methods. In future, we will combine other methods and run with real data.

## Conclusions

ClipCrop is a tool for detcting SVs with soft-clipiing information. Soft-clipped sequences are partially unmatched fragments in a mapped read. ClipCrop remaps these sequence and infers which type of SV events exists from the mapping pattern. ClipCrop can detect SVs with higher discovering rate and call accuracy than any other tool in simulation data set, especially in short size duplications and insertions. In addition, as ClipCrop does not require a large depth of coverage or long read lengths, it can handle most of current NGS data. Currently, the implementation of ClipCrop is only available in our environment, and we are in the process of deploying. We provide current implementation if you contact us.

## Competing interests

The authors declare that they have no competing interests.

## Authors' contributions

SS conceived the project and design, implemented the algorithms and simulators, generated simulation data, and performed the computational analysis. TY helped running other SV-detecting tools for comparison. YS made the concept of mapping soft-clipped sequences. SM and MN critically revised the manuscript.
